# 2667. Bridging the Gap: Sexually Transmitted Infections Testing, Treatment, and Prevention at a Miami Syringe Services Program for Women Who Inject Drugs

**DOI:** 10.1093/ofid/ofad500.2278

**Published:** 2023-11-27

**Authors:** Tara E Herrera, Monica Faraldo, David Forrest, Belén Hervera, Katrina Ciraldo, Hansel Tookes, Teresa A Chueng

**Affiliations:** Jackson Memorial Hospital, Miami, Florida; University of Miami Miller School of Medicine, Miami, Florida; University of Miami, Miami, Florida; University of Miami, Miller School of Medicine, Miami, Florida; University of Miami Miller School of Medicine, Miami, Florida; University of Miami Miller School of Medicine, Miami, Florida; University of Miami Miller School of Medicine, Miami, Florida

## Abstract

**Background:**

Women who inject drugs (WWID) remain understudied despite their compounded vulnerability to HIV. WWID experience increased mortality and multilevel barriers to care, including violence, homelessness, stigma, and competing priorities of childcare. In 2016, Florida passed the Infectious Disease Elimination Act (IDEA), legalizing IDEA Syringe Services Program (SSP) as the first in the state. Located in one of the country’s highest-incidence areas of HIV, IDEA SSP in Miami offers harm reduction and health opportunities outside of the traditional health care system. Previous IDEA SSP data show that WWID, representing 27% of participants overall, are more likely to report opioid injection, syringe sharing, injecting more than 5 times a day, sexual activity in the past 30 days, and transactional sex compared to their male counterparts.

**Methods:**

IDEA SSP offered sexually transmitted infection (STI) testing and treatment July 1, 2022 through March 31, 2023. This descriptive study included women of childbearing age (18-44 years old) who injected drugs and were SSP participants (N=47).

**Results:**

Among chlamydial infections, 2.1% had oropharyngeal, 4.3% had urethral, and 6.4% had rectal involvement. Three women (6.4%) experienced both gonococcal pharyngitis and urethritis. Rectal gonorrhea was detected in four of the 47 women (8.5%), with half testing positive twice within a 3-month period. 14 women (29.8%) demonstrated reactive syphilis testing.

Of 37 women consenting to testing, three screened positive for pregnancy (8.1%). One had gonococcal pharyngitis, proctitis, and urethritis and two had other STI co-infections.

Within this cohort of 47 women, 31 of the 32 HIV-negative participants initiated pre-exposure prophylaxis (PrEP) of HIV at IDEA SSP. Of the 12 with HIV, 83% reported adherence to antiretroviral therapy (ART). Three women had unknown HIV status.

**Conclusion:**

Despite high HIV/STI co-infection and stigma precluding care in traditional health settings, WWID accessing IDEA SSP engaged with PrEP/ART and STI screening, including 100% of women receiving treatment for gonorrhea, chlamydia, and syphilis. Tailoring STI services at an SSP where WWID access nonjudgmental care caters to a historically neglected but key cohort in Ending the HIV Epidemic.

**Disclosures:**

**Hansel Tookes, III, MD, MPH**, Gilead sciences: Grant/Research Support|Viiv: Grant/Research Support

